# Parasitological Study on Fruits Sold in Huye Complex Market and Rango Local Market in Rwanda

**DOI:** 10.24248/eahrj.v8i1.766

**Published:** 2024-03-28

**Authors:** Eric Murinda, Ally Dusabimana

**Affiliations:** a Department of Biomedical Laboratory Sciences (BLS), Faculty of of science and Technology Catholic University of Rwanda

## Abstract

**Background:**

Fruits are essential for good health and they form a major component of human diet. They are vital energy contributors that are depended upon all levels of human as food supplement or nutrients. Although they have all these benefits, when there are not handled with good hygiene they can transmit parasitic infections especially intestinal parasitic infections in the world including Rwanda. The study was conducted to determine the parasitological patterns on fruits purchased in “Huye complex market and Rango local market” in southern province of Rwanda.

**Objective:**

To assess and identify the parasitological patterns on fruits purchased in Huye complex market and Rango local market in Southern Province of Rwanda.

**Methodology:**

A cross-sectional study was designed and 188 fruits were sampled from Huye complex market and Rango local market then washed by using normal saline and the suspension was centrifuged and the sedimentation was examined on a microscope. Data was analysed using Statistical Package for Social Sciences (SPSS) version 23.0 and MS Excel.

**Results:**

The overall prevalence of all parasites obtained from the fruits was 52.65%, whereby the prevalence of all parasites in Rango local and Huye complex markets was 66.63% and 44.7%, respectively. The frequency of identified parasites' contamination was *Ascaris Lumbricoides* 44.44%, cysts of *Giardia lamblia* 24.24%, eggs of *Trichuris trichura* 10.1%, cysts of *Entamoeba coli* 17.17% and *Entamoeba histolytica* 4.04%.

**Conclusion:**

The level of fruits contamination by pathogenic parasites remain high, hence regular health education on hygiene of fruit have to be increased to the population and continuous monitoring on sellers of fruits is required.

## BACKGROUND

Vegetables and fruit are extremely important in human nutrition as sources of nutrients and nonnutritive food constituents as well as for the reduction in disease risks. While their importance as sources of nutrients and non-nutritive food constituents is generally accepted, there are still uncertainties regarding their relevance for the prevention of diseases.^1^ Fruits particularly those eaten raw and without peeling can be agent of transmission of protozoa and helminthes.^2^ Poor hygiene of fruits is a potential source of human parasitic infection by contamination during production, harvesting, collection, transport and preparation or processing. The most common species for human intestinal parasitic infections are *Ascaris lumbricoides*, *Trichuris trichiura*, *Ancylostoma duodenale*, *Nectar americanus*, *Balantidium coli*, *Giardia intestinalis* and *Blastocystic hominis* which are which are commonly found in unhygienic environments.^3^

In developing countries, because of inadequate or even nonexistent systems for the routine diagnosis of food-borne pathogens, most disease outbreaks caused by contaminated vegetables go undetected, and the incidence of food-borne pathogens in food is underestimated.^4^ An important nutritional value of fruits is its antioxidant contents. Some fruits such as orange, carrot, garden egg and tomato have the highest antioxidant value.^5^ In Africa, the transmission of intestinal parasitic infection has been considered to increase due to the frequent use of untreated human or animal dung as manure in cultivation by local farmers, which serve as a source of zoonotic parasitic infections.^6^ However, fruits and vegetables, especially, those that are consumed raw and or not properly washed, have been the major source of transmission of human pathogens.^7^

All over the world, vegetables and fruits are eaten raw or cooked lightly to preserve flavor and this practice may very often lead to the food-borne parasitic infections in humans.^8^ An estimated 600 million people, almost 1 in 10 people in the world, fall ill annually for consuming contaminated food, with diarrheal disease being the most common form of these illnesses resulting in the loss of 33 million healthy life years (DALYs). About US$ 110 billion is lost each year in productivity and medical expenses resulting from unsafe food in low- and middle-income countries.^9^ Intestinal parasitic infection may be acquired in different ways.^10^ Previous studies have revealed that many types of fruits purchased at markets in different regions from many non-developing and developing countries were contaminated with helminthes eggs as well as protozoan oocysts.^11^

The rate of contamination and species of contaminant parasites varies based on climatic, ecological, and human factors. Therefore, local data about the contamination status and predisposing factors augments efforts for successful control of parasitic diseases.^12^ It has been reported that 13.5 % of the vegetables sold at central markets in Khartoum were microscopically positive for intestinal parasites.^13^ In Ethiopia, the presence of foodborne pathogens (parasites and bacteria) is significant and is considered as One Health concern.^14^ Out of the 105 fruit and vegetable samples bought from three selected markets in Abuja metropolis, 35 (33.3%) were contaminated with at least a single parasite species.^15^

Several studies on the contamination of fruits, vegetables and nuts have been documented in different parts of the world. About 19.5% of fruits and vegetables in a study conducted in Kaduna, Nigeria were contaminated with at least one parasitic helminth out of the four species; *Strongyloides stercoralis* (51.9%); *Fasciola sp* (27.8%); *A. lumbricoides* (11.3%) and Hookworm (9.0%).^16^ Eating unclean, raw or undercooked fruits and vegetables, propagates transmission of intestinal parasitic infections.^17^ Fruits have played a great role in the human health and today they are highly purchased in the markets and streets as well. Some of those fruits are sold in a packaged way that the buyer does not need to wash or cut the fruits before eating. Most of those vendors are inexperienced on personal hygiene matters.^18^ Therefore, this study was conducted to assess and identify the parasitological patterns and contamination status on fruits purchased in Huye complex market and Rango local market in southern province of Rwanda.

## METHODS

### Study Design

The study design was cross-sectional, data were collected from vendors of fruits to be sold in two markets i.e., “Huye complex market and Rango local market” from Monday to Friday, in a period of 3 months spanning from 01 October up to 31 December 2019.

### Population and Sample Size Estimation

Four different types of fruit (avocados, banana, passion and mango) are the most obtained fruits in the 2 markets, 12 vendors accepted to sell their fruits at a fair cost, 6 from Rango local markets and 6 from Huye complex markets. A simple random estimation was used to choose the sample size of 188 fruits from different vendors.

### Sample Collection

A total of 188 different ffruits were collected from 4 types of fruits that are mostly available in the 2 markets, 94 different types of fruits (24 for avocadoes, yellow bananas and 23 for passion and oranges) from Huye complex market and 94 different types of fruits (24 for avocadoes, yellow bananas and 23 for passion and oranges) from Rango local market and a cross-sectional study design was used.

The samples were collected in sterile plastic bags bought from health facility, and each type of fruit was labeled and placed in its own plastic bag according to the corresponding fruit type.

### Study Area

Rango local market is located in Tumba sector with 31,399 people, Huye district, southern province in Rwanda. It was built in 1997 and refurbished in 2019. People from Tumba sector and other nearby sectors use these markets to buy and sell various farm produce. Huye complex market also is located in Huye district, and it is the biggest market in this district. All vendors from 14 sectors of Huye district bring their fruits at this market.

### Sample preparation and Procedure

After fruits sample collection, a single type of each fruit was washed with a saline solution (0.9% NaCl) and then washed in sterile beakers. New gloves were used for each fruit and the washing solution was placed in BenchMate Microcentrifuge tubes and centrifuged at 3000 rpm for 5 minutes, the supernatant was discarded, the sediment was put on slide covered with cover slip and examined under 10x and 40x magnifying objectives of light compound microscope. Microscopic observation was made and the results were recorded for further analysis.

### Data Analysis

Data was analysed using Microsoft excel and Statistical Package for Social Sciences (SPSS) version 23.0.

### Inclusion Criteria

Any of the fruits that vendors brought to Huye complex market and Rango local market during the period of the study were included after requesting vendors to sell to us at a fair price.

### Exclusion Criteria

Exclusion criteria was those fruits vendors who did not accept to participate in the present study.

### Ethical Consideration

The study was conducted after obtaining ethical clearance from Catholic University of Rwanda Research Ethical Committee and Rango Health Center with a reference number: 1090/UCR/RHC/2019. A verbal consent was sought for from the vendors to participate in the study after explaining the purpose of the study. Each vendor was paid for the fruits bought.

## RESULTS

The prevalence of parasitic contamination on fruits sold on both Huye complex market and Rango local market are presented in Table 1 shows 47.34% of fruits that were negative for parasitic contamination while 52.66% were positive with contamination by a wide variety of parasites including *Ascaris lumbricoides, Giardia lamblia, Trichuris trichuria, Entemoeba coli, Entamoeba histolytica.* This was identified in a number of fruits including avocadoes, bananas, passion and oranges among other fruits sold in the markets.

**TABLE 1:** The Prevalence of Parasitic Contamination on Fruits sold on both Huye Complex Market and Rango Local Market

Number of fruits examined	Number of fruits without parasitic contamination (Negative sample)	Number of fruits with parasitic contamination (positive samples)
188	89	99
Percentage	47.34%	52.66%

The comparison of the level of parasitic contamination between fruits sold in Huye complex market and Rango local market is summarized in Table 2. The sample of fruits examined was 94 from each market accounting for 50% of the total sample. From Rango local market, 37 fruits (39.36%) were negative while 57 samples (60.63%) were positive for one or many of the parasites listed above. On the other hand, Huye complex market 52 fruits (55.3%) were negative while 42 (44.7%) were positive for one or many of the parasites listed above. This indicates a visible gap between the markets whereby there is more parasitic contamination in fruits sold in Rango local market than Huye complex market.

**TABLE 2:** : The Comparison of the Level of Parasitic Contamination between Fruits Sold in Huye Complex Market and Rango Local Market

Rango local market			Huye complex market		
Number of fruit examined	Number of negative samples	Number of positive samples	Number of fruits examined	Number of negative samples	Number of positive samples
94	37	57	94	52	42
Percentage	39.36%	60.63%	Percentage	55.3%	44.7%

The frequency of parasites identified on fruits sold in Huye Complex Market and Rango local market and frequency of parasites on each type of fruits examined is shown in Table 3. *Ascaris lumbricoides* was the most prevalent of all the parasites which were identified with 16.16% of avocadoes, 14.14% of banana, 6.06% of passion and 8.08% of oranges amounting to a total of 44.44% of all samples. *Giardia lamblia* was obtained with 7.07% on avocadoes, 8.08% on banana, 4.04% on passion and 5.05% on oranges resulting in a total of 24.24% of all samples. *Entamoeba coli* contaminating 6.06% of avocadoes, 4.04% of banana, 1.01% of passion fruits and 6.06% of oranges rounding into 17.17% of all samples. *Trichuris trichiura* was identified in 2.02% of avocadoes, 2.02% of banana, 4.04% of passion fruits and 2.02% of oranges representing 10.1% of all total sample. *Entamoeba histolytica* was abtained on 3.03% of avocadoes, 1.01% on banana, while none was seen in either passion or oranges; this resulted into 4.04% of the total sample from all markets.

**TABLE 3:** : The Proportions of Parasites Identified on each Type of Fruits Sold in Huye Complex Market and Rango Local Market

	Avocadoes	Banana	Passion fruits	Oranges	%
Ascaris lumbricoides	16.16%	14.14%	6.06%	8.08%	44.44%
Giardia lambria	7.07%	8.08%	4.04%	5.05%	24.24%
T. trichiura	2.02%	2.02%	4.04%	2.02%	10.1%
Entamoeba coli	6.06%	4.04%	1.01%	6.06%	17.17%
Entamoeba histolytica	3.03%	1.01%	0%	0%	4.04%

## DISCUSSION

In this study the level of fruits contamination in Rango local market and Huye complex market was obtained by comparing fruits sold in the two markets. The level of contamination was higher in Rango local market (60.63%) when compared to Huye complex market (44.7%). This significant difference could be because Rango local market is located in a rural area with poor hygiene, unlike Huye complex market which is located in the city. A similar finding was reported by a study conducted in Ethiopia^19^, which found the level of fruits contamination to be 57.8%. The slight difference between the two studies is attributed to the different techniques used, whereby this present study used concentrated saline samples while the study of Tefera used zinc sulfate flotation and formal ether concentration technique for analysis of study subjects.

The present study demonstrates higher rates of parasitic contamination on fruits than that reported in a previous study done in Ibadan town where a total of 96 samples were examined for intestinal parasites using sedimentation and floatation methods where 34 (35.4%) of the 96 fruits were positive for intestinal parasites microscopically. ^20^ The present study findings are also higher compared to the study done in Bauchi, Nigeria where the rate of 14.3% was obtained in 4 different types of fruits using a screening by simple floatation and formol-ether concentration technique.^11^ Post-harvesting handling methods of fruits and transport method employed, contamination during processing, distribution and marketing and ineffective hygienic practice by famers and vendors could be the reason for variation in prevalence in the present study in Rango local market compared to Huye complex market.

This study has found the following parasites to be the most frequently contaminants, *Ascaris lumbricoides*, *Giardia lamblia*, *Trichuris trichura, Entamoeba coli* and *Entamoeba histolytica*. The most prevalent parasite in the present study was *A. lumbricoides* which is similar to the study done in Akure Metropolis, Ondo State, Nigeria.^21^ The present study findings are also similar to those of a study done in Bauchi, Nigeria where *A. lumbricoides* was the most prevalent parasite in banana fruits.^11^ This seems to be due to the spread of the parasite especially in poor sanitary conditions and also due to frequent use of untreated human or animal dung as manure in cultivation and differences in illiteracy between the regions.

Detection of some parasites such as *Ascaris lumbricoides, Trichius trichiura* and *Giardia lamblia* in the present study samples demonstrates poor hygienic standard in the studied area on the avocados, banana, passion and mango fruits.

## CONCLUSION

The results from this research shows contamination level of fruits with intestinal parasites, indicating presence of a great risk of acquiring intestinal parasite infections by eating improperly washed fruits and may have serious public health implications. Health education with respect to personal hygiene and implementing appropriate measures to help people in prevention and eradication of intestinal parasitic infections are the most practical and useful approach in order to achieve the desired control in the studied area. The local people must be effectively trained in proper washing of the fruits prior to consumption.

### Limitations of this study

Fruits in this study were from only two markets Huye complex market and Rango local market while there are more than 500 markets all over the country therefore the prevalence estimates cannot be generalized for the whole country.
